# Supplementary material for: The genome sequence of the Field Cuckoo-bee, Bombus campestris (Panzer, 1801)
Source: Wellcome Open Res. 2023 Feb 15;8:77. [Version 1] doi: 10.12688/wellcomeopenres.18985.1 (PMC10646345; doi:10.12688/wellcomeopenres.18985.1)
Supplement: Peer review history

# Reviewer report: Reviewer response for version 1

**DOI:** 10.21956/wellcomeopenres.21048.r68990

**Published:** 2023-11-14

**Authors:** Richard P Meisel

**Affiliations:** 1 University of Houston, Houston, Texas, USA

Crowley describes the de novo sequencing, assembly, and annotation of the Bombus campestris genome. The assembly contains 25 "chromosome-level scaffolds". Completeness of the genome assembly was assessed with BUSCO metrics. It would be helpful to know if there are any cytological data on the chromosome number in this species. In other words, is the number of chromosomes in the assembly (25) equal to what is expected based on the actual number of (haploid) chromosomes in this species?

Are sufficient details of methods and materials provided to allow replication by others?

Yes

Is the rationale for creating the dataset(s) clearly described?

Yes

Are the datasets clearly presented in a useable and accessible format?

Yes

Are the protocols appropriate and is the work technically sound?

Yes

Reviewer Expertise:

Evolutionary genetics and genomics

I confirm that I have read this submission and believe that I have an appropriate level of expertise to confirm that it is of an acceptable scientific standard.

# Reviewer report: Reviewer response for version 1

**DOI:** 10.21956/wellcomeopenres.21048.r66950

**Published:** 2023-09-29

**Authors:** Cheng Sun

**Affiliations:** 1 College of Life Sciences, Capital Normal University, Beijing, China

This study provided a high-quality reference genome for the field cuckoo bumblebee, which will be useful for understanding the evolution of social parasitism and the conservation of this species. In general this is a well-organized manuscript, and I only have the following suggestions:

1. Double check the number of genes in hymenoptera_odb10 reference set. It should be 5991 other than 5911.
2. It will be better to use a color different from red to show the border of scaffold in Figure 5. For me, it's hard to count how many scaffolds there are.

Are sufficient details of methods and materials provided to allow replication by others?

Yes

Is the rationale for creating the dataset(s) clearly described?

Yes

Are the datasets clearly presented in a useable and accessible format?

Yes

Are the protocols appropriate and is the work technically sound?

Yes

Reviewer Expertise:

bumblebee genome evolution

I confirm that I have read this submission and believe that I have an appropriate level of expertise to confirm that it is of an acceptable scientific standard.
